# Bronchial Fistula: Rare Complication of Treatment with Anlotinib

**DOI:** 10.3779/j.issn.1009-3419.2020.102.40

**Published:** 2020-10-20

**Authors:** Pengbo DENG, Chengping HU, Yuanyuan LI, Liming CAO, Huaping YANG, Min LI, Jian AN, Juan JIANG, Qihua GU

**Affiliations:** 1 Department of Respiratory Medicine, Xiangya Hospital, Central South University, Changsha 410008, China; 2 Xiangya Lung Cancer Center, Xiangya Hospital, Central South University, Changsha 410008, China

**Keywords:** Anlotinib, Lung neoplasms, Bronchial fistula, Broncho-pericardial fistula, Broncho-pleural fistula, Esophago-tracheobronchial fistula

## Abstract

**Background and objective:**

Anlotinib is a newly developed small molecule multiple receptor tyrosine kinase (RTK) inhibitor that was approved for the treatment of patients with lung cancer in China. We aim to report 3 cases of rare complication of anlotinib-bronchial fistula (BF) during the treatment of lung cancer patients and summarize the possible causes.

**Methods:**

We collected three patients who developed BF due to anlotinib treatment, and conducted a search of Medline and PubMed for medical literature published between 2018 and 2020 using the following search terms: "anlotinib, " "lung cancer, " and "fistula."

**Results:**

Our literature search produced two case reports (three patients) which, in addition to our three patients. We collated the patients' clinical characteristics including demographic information, cancer type, imaging features, treatment received, risk factors for anlotinib related BF, and treatment-related outcomes. The six patients shared some common characteristics: advanced age, male, concurrent infection symptoms, diabetes mellitus (DM), advanced squamous cell and small cell lung cancers, centrally located tumors, tumor measuring ≥5 cm in longest diameter, and newly formed tumor cavitation after multi-line treatment especially after receiving radiotherapy. Fistula types included broncho-pericardial fistula, broncho-pleural fistula, and esophago-tracheobronchial fistula. Six patients all died within 6 months.

**Conclusion:**

Although anlotinib is relatively safe, it is still necessary to pay attention to the occurrence of BF, a rare treatment side effect that threatens the quality of life and overall survival of patients. Anlotinib, therefore, requires selective use and close observation of high-risk patients.

## Introduction

The application of anti-tumor angiogenesis and growth drugs in the treatment of lung cancer is widely recognized. Anlotinib is a newly developed small molecule multiple receptor tyrosine kinase (RTKs) inhibitor for oral medication that targets vascular endothelial growth factor receptor (VEGFR) 2/3, platelet-derived growth factor receptors (PDGFR) α/β, fibroblast growth factor receptors (FGFR) 1-4, c-Kit and Ret^[1]^, and has inhibitory effects on tumor angiogenesis. It was approved by China Food and Drug Administration (CFDA) for the treatment of patients with both non-small cell lung cancer (NSCLC) and small cell lung cancer (SCLC) and progression after > 2 lines of chemotherapy. In December 2015 and June 2016, anlotinib was certified by the US Food and Drug Administration (FDA) for the treatment of ovarian cancer and soft tissue sarcoma.

Although anlotinib has fewer side effects than other small molecule drugs that target vascular function and inhibit tumor angiogenesis, it still has a 20% incidence of grade 3 or 4 treatment-related adverse events, and a dose reduction or suspension of treatment is usually required^[2, 3]^. The complications of bronchial fistula (BF) are not stated in clinical trials and CFDA labeling, however, we have identified in our clinic several cases of patients with post-anlotinib treatment-related BF, from which three cases were selected and combined with three cases reported by others, and their patient characteristics collated and analyzed to guide future clinical applications of anlotinib.

## Materials and Methods

### Case presentation

Case 1: A 69-year-old Chinese male smoker presented to the local hospital with a persistent dry cough for nine months, hoarseness for two months, and shortness of breath for four days. A computed tomography (CT) scan of the chest showed that the left main bronchus was thickened and occluded, and the left hilar was occupied by a tumor (28 mm×31 mm) associated with the left pulmonary artery and invasion of the left atrium (Fig 1A). Electron microscopy revealed a left bronchial lesion, and its biopsy revealed a squamous cell carcinoma. Electron microscopy guided biopsy of the left bronchial lesion provided a histopathological diagnosis of squamous cell lung cancer in July 2018. The patient refused chemotherapy and was instead treated with anlotinib (orally, 12 mg once daily on day 1 to 14 of a 21-day cycle). After the use of anlotinib for 1.5 mon, the patient's symptoms improved, and a CT scan identified possible cavitation in the left upper lung tumor (Fig 1B). However, another 1.5 months later, the patient worsened and displayed the following: shortness of breath, CT indicating the upper left lung tumor was larger than the before (55 mm×35 mm), with cavitation, as well as communication with the left main bronchus and pericardial cavity with large amounts of gas buildup, suggesting the formation of a possible broncho-pericardial fistula (BPCF) (Fig 1C). A second bronchoscopy showed a huge fistula at the left main bronchus, a huge cavity was seen with a visible heartbeat on the inner wall (Fig 1D-Fig 1F). The patient was treated with anti-infectives, given nutritional supplementation, and gas drained through a catheter inserted into his pericardium for 5 d, after which his CT showed a significant reduction of gas and his pericardium was partially conglutinated (Fig 1D). The patient, who was in poor condition, was required to return to the local hospital for supportive treatment, and eventually died due to tumor progression 2 months later.

**1:**
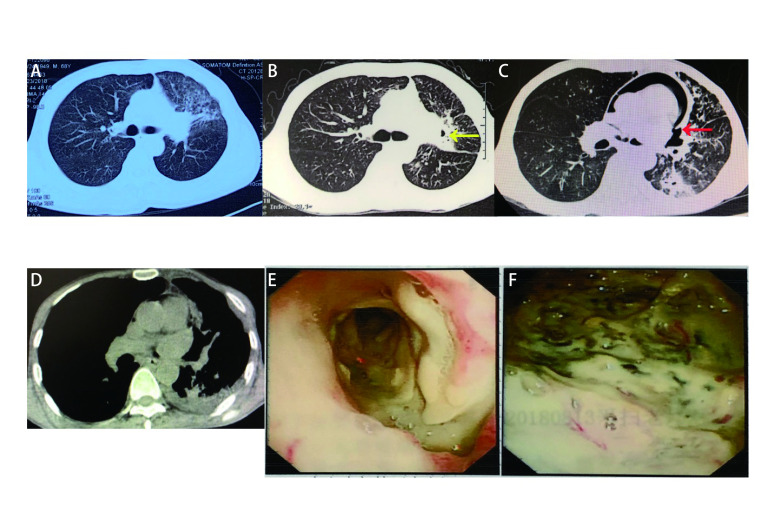
Broncho-pericardial fistula (BPCF) appears in a patient with pulmonary squamous cancer after using anlotinib. A: Left hilar mass (28 mm×31 mm), before use of anlotinib; B: After use of anlotinib for 1.5 mon, a cavity was formed in the left upper lung tumor (yellow arrow: cavity); C: A fistula communicating with the left main bronchus and pericardial cavity with large gas buildup in it, suggesting BPCF (red arrow); D: After drained gas with catheter stetted into pericardium for 5 days, computed tomography showed significant reduction of gas and pericardium partially conglutinated; E: (Bronchoscopy) A huge fistula at the left main bronchus; F: (Bronchoscopy) A huge cavity was seen with heart beat visible on the inner wall.

Case 2: A 63-year-old Chinese male smoker diagnosed with squamous cell cancer of the right lung [epidermal growth factor receptor (EGFR) 19del, stage IVb] in 2016, with a history of diabetes mellitus (DM) (poorly controlled), who successively received the following: four series of chemotherapy cycles with gemcitabine (GEM)+carboplatin (CBP); four months of targeted therapy (icotinib); 36 Gy (3 Gy×12 fractions) sequential radiation therapy on the lung tumor and mediastinal lymph node metastasis; and two chemotherapy cycles with paclitaxel (PTX); was started on anlotinib (orally, 12 mg once daily on day 1 to 14 of a 21-day cycle) in 2018 for four months. Before initiating anlotinib, the patient's CT scan revealed a tumor in the central right upper lung (55 mm×65 mm) with an invasion of the right main bronchus and obstructive pneumonia in the right upper lung (Fig 2A). The patient's symptoms were alleviated transiently, and his CT revealed huge cavitation in the upper right lung tumor (Fig 2B). After four months of anlotinib treatment (in October 2018), the patient worsened and began coughing pyohemosputum (100 mL/d), blood sputum and fever, and his CT displayed a right broncho-pleural fistula (BPF) with liquid pneumothorax formation (Fig 2C). *Staphylococcus aureus* and *stenotrophomonas maltophilia* were cultured in the patient's sputum, and *Actinomyces cariestus* was cultured in the pleural drainage fluid. The patient received closed thoracic drainage and multiple anti-bacterial therapies, after which the fever and drainage decreased. However, the patient died two months later due to tumor progression, failure of the fistula to close, and worsened infection.

**2:**
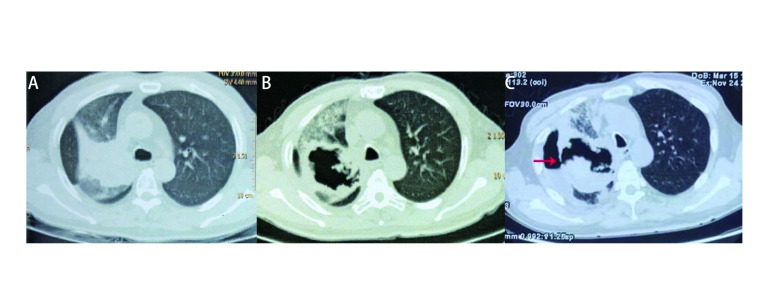
Broncho-pleural fistula (BPF) appears in a patient with pulmonary squamous cancer after using anlotinib. A: Central right upper lung occupied mass (55 mm×65 mm) with the right main bronchus invasion and right upper lung obstructive pneumonia, before use anlotinib; B: A huge cavity formed in the upper right lung mass after 4 mon of use of anlotinib; C: Right BPF (red arrow) with liquid pneumothorax formation.

Case 3: A 49-year-old Chinese male smoker complained of dry cough for three months. A CT scan showed an upper left lung tumor (92 mm×89 mm) with left upper lung atelectasis, along with mediastinal lymphadenopathy (short axis=46 mm), and multiple thin-walled cavitation lesions in the left lower lung considered to be lung cancer metastases (Fig 3A, Fig 3B). Bronchoscopy showed an endobronchial lesion in the left upper lobe bronchus, with an invasion of the left lower lobe bronchus and the left main stem. The patient was diagnosed in December 2019 with small cell lung cancer by endobronchial biopsy and confirmed to have multiple metastases to the pleura, pericardium, liver, and spleen. Meanwhile, the patient was also diagnosed with active viral hepatitis B and poorly controlled DM. The patient received anti-tumor treatment with etoposide (VP-16), CBP, plus anlotinib (orally, 12 mg once daily on day 1 to 14 of a 21 d cycle) while receiving blood glucose regulation and anti-HBV treatment. Ten days after completing his first course of treatment, the patient suddenly developed left chest pain, increased shortness of breath, cough, and sputum, without fever. Due to the emergence of the coronavirus disease 2019 (COVID-19) epidemic in China, he did not go to the hospital for examination promptly and continued to insist on oral treatment with anlotinib at home. Six weeks later, his CT scan depicted smaller left upper lung tumor and mediastinal lymph nodes but also showed left lower lung atelectasis and a worm-like cavity, with an incomplete cavity wall and communication with the pleural cavity that led to the formation of a liquid pneumothorax (Fig 3C). Closed thoracic drainage was completed and a large amount of purulent fluid and gas were drained, which was an indication of BPF combined with pneumothorax. *Klebsiella pneumoniae* was also cultured in the pleural drainage fluid. The patient died 2.5 months later due to tumor progression and worsened infection.

**3:**
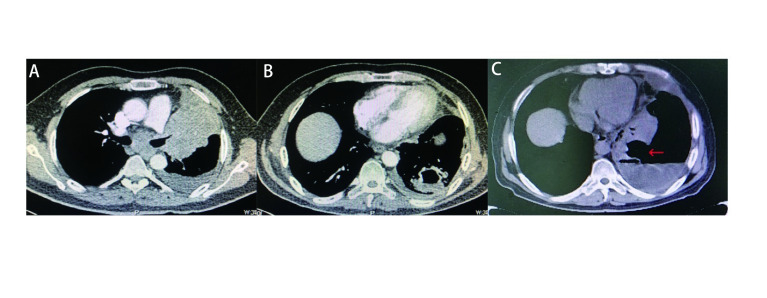
BPF appears in a patient with small cell lung cancer after using anlotinib. A: Upper left lung mass (92 mm×89 mm) with left upper lung atelectasis, along with mediastinal lymphadenopathy (short axis=46 mm), before use anlotinib; B: Thin-walled cavitation lesion in the left lower lung considered to be lung cancer metastases, before use anlotinib; C: Left lower lung atelectasis with a cavity wall incomplete and communicates with the pleural cavity, indicating broncho-pleural fistula (BPF, red arrow), which led to the formation of liquid pneumothorax.

### Review criteria

We conducted a detailed search of the literature (written in English) published between 2018 and 2020 in Medline and PubMed using the following search criteria: "anlotinib, " "lung cancer, " and "fistula". The search retrieved two case reports of three lung cancer patients with fistulas related to the use of anlotinib. We read the full texts, summarized and analyzed the general information, tumor-related data and combined treatment of these three patients, in addition to data from our three patients, and tried to derive some meaningful conjectures.

## Results

The patients' demographic information, cancer type, imaging features, treatment, risk factors of anlotinib related BF and outcome, *etc*. are shown in Tab 1^[4, 5]^. Among the six cases in this review (including our case report), the mean age was 59.7-year-old (ranging from 49 yr to 69 yr), and 83.8% (5/6) of them are over 50 yr. The ratio of men to women was 5:1. Smoking and non-smoking patients were equally divided (ratio 3:3). In our cases, two of the three patients had poorly controlled DM, and three had concurrent symptoms of infection when the bronchial fistula developed, including fever (2/3) and purulent sputum (3/3). Five patients (83.3%) were diagnosed with squamous cell NSCLC and one (16.7%) was diagnosed with SCLC, all (100.0%) of them were central type. All patients (100.0%) had reached stage IIIb and above at the start of anlotinib treatment, five of whom (83.3%) were in a metastatically advanced stage. The tumors on the CT showed just one case had a tumor with a < 5 cm longest diameter, while the others were ≥5 cm (83.3%). Before anlotinib treatment, only one patient had tumor cavitation, however, after anlotinib treatment, three more patients formed new cavities, with cavity formation rate reached 66.7%. The tumor of the majority of the patients (66.7%, 4/6) measured less than 1 cm from the pleura, except for two patients with esophago-tracheobronchial fistula (ETBF). The number of patients treated with anlotinib in 1^st^, 2^nd^ and 3^rd^ line treatment (or greater) was two, one, and three respectively, indicating 83.3% (5/6) patients received ≥2 line treatment. 33.3% (2/6) patients had received thoracic radiation therapy (TRT). The types of bronchial fistulas the six patients developed were BPF (three patients), ETBF (two patients), and one extremely rare BPCF. After the emergence of BF, all patients (100.0%) died within six months, with an average survival time of 2.67 months (ranging from 0.5 mon-6 mon).

**1:** General characters of the case

Article	Gender	Age	Smoking history	Hemopt-ysis	Cancer type	Staging	Location of mass	Tumor diameter	Cavity		Diistance of the mass from the pleura	Treatment	Use of anlotinib in which line of treatment	Type of fistula	Other risk factors of anlotinib related BF	Outcome
									Before anlotinib	After anlotinib						
Our case	M	69 yr	Yes	Yes	NSCLC (squamous cell)	IVa	Central	≥5 cm	No	Yes	0 cm (Mediastinal pleura)	anlotinib×3 m	1^st^ line	BPCF	DM, infection	Died in 2 mon
	M	63 yr	Yes	Yes	NSCLC (squamous cell)	IVb	Central	≥5 cm	No	Yes	1 cm	GEM+CBP×4, icotinib×4 m, TRT (36 Gy), PTX×2, anlotinib×4 m	5^th^ line	BPF	Infection	Died in 2 mon
	M	49 yr	Yes	No	SCLC	IVc	Central	≥5 cm	Yes	Yes	0.5 cm	VP-16+CBP×1 +anlotinib×1.3 m	1^st^ line	BPF	DM, infection	Died in 2.5 mon
Zhang PL, 2019	F	55 yr	No	No	NSCLC (squamous cell)	IIIb	Central	≥5 cm	No	No	Unknown	PTX-liposome+CDDP×6, anlotinib×1 m	2^nd^ line	ETBF	Unknown	Died in 3 mon
	M	53 yr	No	Yes	NSCLC (squamous cell)	IV	Central	< 5 cm	No	No	Unknown	GEM+NDP×6, TRT(45 Gy), nanoparticle albumin-PTX+NDP×6, anlotinib×1 m	4^th^ line	ETBF	-	Died in 6 mon
Li D, 2019	M	69 yr	No	Yes	NSCLC (squamous cell)	IV	Central	≥5 cm	No	Yes	0 cm	GEM+CDDP×4, PTX-liposome+NDP×6, GEM+NDP×2, anlotinib×1.3 m	4^th^ line	BPF	-	Died in 0.5 mon
M: male; F: female; NSCLC: non-small cell lung cancer; SCLC: small cell lung cancer; BPCF: broncho-pericardial fistula; BPF: broncho-pleural fistula; ETBF: esophago-tracheobronchial fistula; TRT: thoracic radiation therapy; CDDP: cisplatin; CBP: carboplatin; NDP: nedaplatin; VP-16: etoposide; PTX: paclitaxel; GEM: gemcitabine; DM: diabetes mellitus.																

## Discussion

Anlotinib is a novel orally administered multi-RTK inhibitor that inhibits tumor angiogenesis and growth^[6]^, which was demonstrated to have manageable toxicity, longer circulation times and broad-spectrum anti-tumor potential^[7]^. Anlotinib was approved by CFDA as a candidate drug for third-line treatment of advanced NSCLC on May 9, 2018, and for third-line treatment of SCLC on September 4, 2019. The above is based on a randomized double-blind controlled phase III clinical trial called ALTER0303, whose results confirmed that anlotinib as a third-line treatment for NSCLC patients can significantly improve their overall survival rate with controllable side effects^[2]^. Another randomized phase II study called ALTER1202 indicated that anlotinib appeared to provide significant progression-free survival (PFS) and disease control rate (DCR) benefits for patients with SCLC who progressed after two lines of chemotherapy^[8, 9]^.

The above clinical studies found that hypertension, elevated thyroid-stimulating hormone, hand-and-foot syndrome, elevated thyroglobulin, elevated total cholesterol, and diarrhea^[2, 3]^ were frequently observed during treatment with anlotinib. While hypertension, hyponatremia, and hemoptysis are the most common adverse events reported by patients with squamous cell lung cancer^[10]^, BF has not been reported in clinical studies.

BF is a rare, life-threatening condition that seriously affects the quality of life of patients with lung cancer. It includes ETBF, BPF, broncho-mediastinal fistula (BMF), and the extremely rare BPCF. It can be caused by a tumor invasion but is more common in tumor patients after chemotherapy, radiotherapy, and interventional treatments^[11, 12]^. The incidence of malignant ETBF has been reported to be 0.16% to 0.3% for lung cancer^[13, 14]^, with a median survival time (MST) from diagnosis of only 6 weeks in the absence of appropriate treatment^[13]^. BPF is more common in lung cancer patients after surgery, directly caused by lung cancer invasion is rarely reported which usually combined with infection^[15]^. In recent years, the use of anti-tumor vascular targeting drugs has also been found to cause BF. In the earliest clinical studies of bevacizumab, the first anti-angiogenic drug used in clinics, it was found that bevacizumab used in the treatment of lung cancer often caused BF, of which ETBF was the most common^[16]^, BPF and BMF^[17]^ were also have been reported. In phase II clinical trials of bevacizumab in combination with chemotherapy and radiation for the treatment of SCLC and NSCLC, both groups had a very high incidence of BF (2/29, 2/5 respectively), and the death of one patient prompted early trial closures^[18]^. They consider that the unique role of bevacizumab in inhibiting angiogenesis and consequently delaying wound healing were likely accounts for this rare and serious effect. Therefore, as anlotinib has a similar anti-angiogenic effect with bevacizumab, when a patient develops a BF during the treatment of anlotinib, we should carefully consider the correlation between anlotinib and the BF formation.

Generally, we considered that anti-angiogenic drugs can lead to necrotic cavity formation due to ischemia in the center of the tumor. In our review, five patients (83.3%) had a tumor longer than 5 cm in diameter. And four of the five patients with large tumors formed a necrotic cavity after receiving treatment with anlotinib, leading to rupture of the cavity wall after continuous use. Suggesting that we need to be highly vigilant when using anlotinib to treat large tumors, especially when new cavities appear.

However, there were two cases of ETBF that did not appear to display new necrotic cavities, possibly because the trachea is adjacent to the esophagus, similar to the central tumor, so only slight necrosis of the tube wall will form a fistula. Therefore, patients with ETBF may evade typical necrotic cavities after the use of anlotinib. A case of ETBF caused by bevacizumab reported and summarized 9 similar cases, which concluded that TRT had been suggested to be the most significant risk factor for bevacizumab-related ETBF, also including damage to the esophagus and bulky subcarinal lymph node^[16]^. Also, in our two cases, two had bulky subcarinal lymph node, and one had a history of TRT, indicating TRT and bulky subcarinal lymph node, suggesting that these two factors is also related to ETBF, caused by anlotinib. Acquired ETBF should be suspected whenever patients with mediastinal metastasis develop suspicious clinical symptoms, for example, drinking water-induced choking cough, especially coughing up food just eaten. The diagnosis relies on an esophagram, CT, and videofluoroscopy. Fistulas that occur in the bronchi and its distal end may be difficult to plug with a stent implant and an occlude, and the chance of surgical management for advanced lung cancer patients is almost impossible. However, for ETBF, a fully covered self-expandable metallic stent has been demonstrated as a superior alternative treatment owing to its high efficiency, which immediately relieved the patient's symptoms after palliative treatment^[4]^.

Two of the three BPF patients we evaluated had pathogenic bacteria cultured from sputum or drainage fluid, including *Staphylococcus aureus*, *Actinomycetes* and *Klebsiella pneumoniae*, which can all cause necrotizing pneumonia, lung abscess, and empyema, especially in tumor patients with cavities; this may also be one of the important causes of BF. It has been reported that patients with lung cancer have decreased immunity after chemotherapy and an *actinomycetes* infection may finally lead to the occurrence of BPF^[15]^. Therefore, for lung cancer patients who have a lot of purulent sputum after anlotinib treatment, we need to actively identify the pathogens and utilize anti-infective treatments to prevent infections from aggravating tumor necrosis. Meanwhile, two of our three patients have poorly controlled DM, which causes decreased immunity, making them prone to co-infection. As we know, central lung cancer easily causes obstructive pneumonia, and anlotinib causes tumor necrosis. In this situation, once the patients with DM develop pulmonary infections, it will be more difficult to control. This may be one of the possible risk factors for the formation of BF and also needs to be paid attention to.

83.8% (5/6) of our BF patients are over 50 yr, and half of them are over 60 yr, which can be correlated to the higher incidence of lung cancer in people over 50. Also, the incidence of BF is significantly higher in males than in females, and all lung cancer types are squamous cell carcinoma and small cell lung cancer, which was more common in men. From the perspective of cancer type and location, squamous cell carcinoma and small cell lung cancer are more likely to be located at the hilum and easily advance to the mediastinum and trachea, causing an invasion of the airway mucosa, necrosis, and bleeding, leading to the defect of the tracheal wall, and fistulas are also easily formed. Five of them were diagnosed by bronchoscopy which further confirmed that the lesions affected the trachea.

In our review, four of the six patients (66.7%) were undergoing treatment with ≥2 lines of therapy, and three were treated with more than four lines (50%). Multi-line treatment means that the patient has a difficult to control tumor, has an impaired immune system, is in poor general condition, and may have cachexia. These characteristics of patients in advanced tumor stages may be the reason that the tumor is more easily necrotic, less repairable, and more likely to cause secondary infection when treated with anlotinib. Two patients also received TRT, which is a recognized risk factor that can cause BF, especially ETBF and BMF^[19-21]^, suggesting that we need to pay attention to the patient's radiotherapy history before initiating anlotinib therapy.

BPCF in lung cancer patient is extremely rare, only a few cases have been reported^[22]^. In one case, a patient with squamous carcinoma, stage IIIa, acquired BPCF after chemotherapy and TRT, after recovered from BPCF, he continued received TRT and chemotherapy, subsequently treated with bevacizumab and cetuximab. Three months later, the patient reappeared BPCF and died of fatal hemoptysis quickly. In this case, the role of bevacizumab in the recurrence of BPCF needs to be paid attention to. In our patient cases, there was a patient with BPCF, who had the following characteristics which may be the risk factors for BPCF development: the tumor was located in the left hilar, close to the mediastinal pleura and pericardium; the patient had poorly controlled DM; although he was using anlotinib as the 1^st^ line treatment, he was in poor general condition (PS score 3) with cachexia; and a necrotic cavity formed in the tumor after treatment with anlotinib. Based on the patient's previously discussed characteristics, when lung cancer patients have exacerbated shortness of breath and chest tightness during the use of anlotinib, in addition to considering other common causes, it is necessary to pay attention to the possibility of BPCF. Prompt puncture drainage and active anti-infective treatments can promote pericardial adhesion closure, which is important for improving the prognosis of patients.

In general, once a patient develops a BF during the use of anlotinib, the survival time will decrease significantly, and there will no longer be an opportunity for further chemotherapy, radiotherapy, and vascular targeted therapy. Most patients die in a short time due to the hard-to-heal fistulas, recurrent infections that are difficult to control, and tumor progression. It has been reported that an adenocarcinoma lung cancer patient developed a BMF after 1^st^ line chemotherapy, radiotherapy, 2^nd^ Line chemotherapy plus bevacizumab, then used Nivolumab (an immune-checkpoint inhibitor) as follow-up treatment. To heal the BF, the treatment requires sufficient anti-tumor efficacy, low risk of bacterial infection, and low risk of weakening the airway wall integrity. Immune checkpoint inhibition is effective for NSCLC but rarely causes necrosis, is less toxic, as well as less likely to lead to infections compared to cytotoxic drugs^[23-26]^. Nivolumab successfully controlled the BMF and the tumor of this patient and prolonged the survival time of the patient for 28 months^[27]^. Also, if patients have sensitive gene mutations, targeted therapy should be considered as one of the alternatives.

## Conclusion

Although the data provided by anlotinib in current clinical trials indicate it is relatively safe, it is still necessary to pay attention to the occurrence of adverse reactions in clinical applications, such as BF. The incidence of bronchial fistula caused by anlotinib in lung cancer is extremely rare, but it seriously affects the quality of life and overall survival of patients. Therefore, we need to use it selectively and closely observe high-risk patients. It's possible risk factors include: (1) ≥50 years old male, (2) DM and infection, (3) central lung cancer, squamous cell carcinoma or SCLC, (4) advanced stage, (5) long diameter of tumor≥5 cm and cavity formation, (6) multi-line treatment, and (7) thoracic radiation therapy.

## Acknowledgement

This manuscript was supported by National Natural Science Foundation of China (to Pengbo Deng)(No.81502699), National Key R&D Program of China (to Chengping HU)(No.2016YFC1303300); National Key R&D Program of China (to Chengping HU)(No.2018YFC1311900); National Multidisciplinary Cooperative Diagnosis and Treatment Capacity Building Project for Major Diseases (to Chengping HU)(Lung Cancer); Xiangya clinical big data project of Central South University (to Chengping HU)(Clinical Big Data Project of Lung Cancer).

## Authorship contributions

Deng PB, Cao LM and Yang HP conceived and designed the study. Deng PB, Li YY and Jiang J collecting the data. Deng PB, Li M, An J and Gu QH analyzed the data. Deng PB, Li YY, Cao LM and Hu CP provided critical inputs on design, analysis, and interpretation of the study. All the authors had access to the data. All authors read and approved the final manuscript as submitted.

## Ethics approval and consent to participate

Our study has already approved by examination and approval documents for scientific research projects Medical Ethics Committee of Xiangya Hospital, Central South University.
